# Agreement between BMI and body fat obesity definitions in a physically active population

**DOI:** 10.1590/2359-3997000000220

**Published:** 2016-11-07

**Authors:** Luiz Guilherme G. Porto, Rosenkranz M. Nogueira, Eugênio C. Nogueira, Guilherme E. Molina, Andrea Farioli, Luiz Fernando Junqueira, Stefanos N. Kales

**Affiliations:** 1 Harvard T. H. Chan School of Public Health Department of Environmental Health Boston MA USA Harvard T. H. Chan School of Public Health, Environmental and Occupational Medicine and Epidemiology Program (EOME), Department of Environmental Health, Boston, MA, USA;; Universidade de Brasília Faculdade de Educação Física Brasília DF Brasil Universidade de Brasília (UnB), Faculdade de Educação Física e Laboratório Cardiovascular da Faculdade de Medicina, Brasília, DF, Brasil; 2 Universidade de Brasília Faculdade de Educação Física Brasília DF Brasil Universidade de Brasília (UnB), Faculdade de Educação Física; Corpo de Bombeiros Militar do Distrito Federal – CBMDF, Brasília, DF, Brasil; 3 Universidade de Brasília Faculdade de Educação Física Brasília DF Brasil Universidade de Brasília (UnB), Faculdade de Educação Física e Laboratório Cardiovascular da Faculdade de Medicina, Brasília, DF, Brasil; 4 Universitá di Bologna Department of Medical and Surgical Sciences Bologna Italy Universitá di Bologna, Department of Medical and Surgical Sciences (DIMEC), Bologna, Italy;; Harvard T. H. Chan School of Public Health Department of Environmental Health Boston MA USA Harvard T. H. Chan School of Public Health, Environmental and Occupational Medicine and Epidemiology Program (EOME), Department of Environmental Health, Boston, MA, USA; 5 Universidade de Brasília Faculdade de Medicina Brasília DF Brasil Universidade de Brasília (UnB), Divisão de Cardiologia, Área de Clínica Médica, Laboratório Cardiovascular, Faculdade de Medicina, Brasília, DF, Brasil; 6 Harvard T. H. Chan School of Public Health Department of Environmental Health Boston MA USA Harvard T. H. Chan School of Public Health, Environmental and Occupational Medicine and Epidemiology Program (EOME), Department of Environmental Health, Boston, MA, USA

**Keywords:** Body composition, firefighters, cardiorespiratory fitness, sensitivity, specificity, BMI, body fat

## Abstract

**Objectives:**

Body mass index (BMI) is a widely used proxy of body composition (BC). Concerns exist regarding possible BMI misclassification among active populations. We compared the prevalence of obesity as categorized by BMI or by skinfold estimates of body fat percentage (BF%) in a physically active population.

**Subjects and methods:**

3,822 military firefighters underwent a physical fitness evaluation including cardiorespiratory fitness (CRF) by the 12 min-Cooper test, abdominal strength by sit-up test (SUT) and body composition (BC) by BF% (as the reference), as well as BMI. Obesity was defined by BF% > 25% and BMI ≥ 30 kg/m^2^. Agreement was evaluated by sensitivity and specificity of BMI, positive and negative predictive values (PPV/NPV), positive and negative likelihood (LR+/LR-), receiver operating characteristic (ROC) curves and also across age, CRF and SUT subgroups.

**Results:**

The prevalence of obesity estimated by BMI (13.3%) was similar to BF% (15.9%). Overall agreement was high (85.8%) and varied in different subgroups (75.3-94.5%). BMI underestimated the prevalence of obesity in all categories with high specificity (≥ 81.2%) and low sensitivity (≤ 67.0). All indices were affected by CRF, age and SUT, with better sensitivity, NPV and LR- in the less fit and older groups; and higher specificity, PPV and LR+ among the fittest and youngest groups. ROC curves showed high area under the curve (≥ 0.77) except for subjects with CRF ≥ 14 METs (= 0.46).

**Conclusion:**

Both measures yielded similar obesity prevalences, with high agreement. BMI did not overestimate obesity prevalence. BMI ≥ 30 was highly specific to exclude obesity. Because of systematic under estimation, a lower BMI cut-off point might be considered in this population.

## INTRODUCTION

Obesity is an epidemic condition that has grown dramatically over the past 50 years, although some data suggest the rate of growth is now slowing (1-3). Nonetheless, recent studies have shown an increase in obesity prevalence in developing countries similar to what was firstly experienced by developed countries (4). The prevalence of obesity has almost doubled between 1980 and 2008 worldwide and increases have occurred in every world region evaluated by the World Health Organization (5). Considering this pandemic scenario and that obesity is a condition associated with other major comorbidities and public health problems, like diabetes (6) and cardiovascular diseases (7), its identification or diagnosis must be based on reliable, simple, and low-cost tools. Several methods and/or techniques have been used to evaluate body composition and to categorize obesity, either for research or for clinical purposes (8). Skinfold thickness and bioelectrical impedance are commonly used for estimating and monitoring body composition in athletics and occupational screenings. The most accurate technique, although more expensive, is the analysis of body composition by the dual-energy X-ray absorptiometry (DEXA), which is considered to be the gold-standard (9,10). The precision, convenience and cost of these methods vary, so that the test of choice depends on the goal of measurement, time available and resources.

Among this variety of body composition determination methods, body mass index (BMI) is the most widely used and recommended by scientific associations (10,11). One of the best known longitudinal studies that had used BMI to continuously assess obesity prevalence, health-related risks and nutritional status of American adults and children, apart from others outcomes, is the National Health and Nutrition Examination Survey (NHANES) (12). In Brazil, since 2006, the Ministry of Health is conducting similar survey (VIGITEL) with the aim to identify health-related risk factors by phone interviews (13). Like NHANES, VIGITEL also characterizes obesity based on a BMI cut point of ≥ 30 kg/m^2^.

Although some possibilities of misclassification of muscle mass as body fat exist (14), BMI has been widely accepted as an appropriate method to estimate obesity prevalence in the public health and health risks contexts. For the most part, misclassification is a significant concern when body composition is evaluated, as in athletic or in physical performance conditions, where the prevalence of fit people with additional weight due to muscle mass is potentially higher than in the general population. In it this context, is plausible to consider that BMI may overestimate overweight and obesity among athletes and among some working populations that are supposedly more active, such as firefighters and other public safety professions. In order to test this hypothesis, we compared the prevalence of obesity as categorized by BMI (≥ 30 kg/m^2^) or by body fat percentage (> 25%) in a large cohort of military firefighters.

## SUBJECTS AND METHODS

### Experimental approach

We performed a cross-sectional study with data collected from standardized records of the 2009 Brasilia Military Firefighters’ annual physical fitness evaluations. All participants were military career firefighters who worked for the Federal District (Brasilia) Military Firefighter Brigade (CBMDF – Portuguese acronym). The CBMDF includes all fire departments in the state of the Brazilian Federal District, where the capital Brasília is located. All data were originally collected for occupational purposes as part of the mandatory annual physical evaluation for all CBMDF firefighter under 50 years old. Firefighters above 50 years old were excluded from this study because they perform a different physical evaluation. All physical fitness evaluations were completed during May 2009. This study is part of a project focused in Brasilia firefighter’s physical fitness and occupational health: The Brasilia Firefighters Study – BFS.

### Subjects

Among the 4216 available physical fitness evaluation records in the database, 394 (9.3%) were excluded: 212 (5.0%) were women and, additionally, 182 (4.3%) men because of incomplete or data containing biologically implausible values. Therefore, all analyses were performed on the cohort of 3,822 men from the CBMDF who had complete data for all variables under analysis.

The use of the recorded physical evaluation data for research purposes was approved by the University of Brasília Faculty of Health Sciences Ethics Committee on Human Research and an authorization from the CBMDF was properly obtained for this study.

### Physical activity evaluation

The CBMDF physical evaluation includes components of health-related physical fitness (HRPF). For this study we focused on the body composition, cardiorespiratory fitness (CRF) and abdominal muscle endurance components in order to evaluate the overall agreement between BMI and BF% for defining obesity and its variance across different HRPF groups and age categories.

Body composition was assessed by the body fat percentage (BF%), as the reference method, using the Guedes 3-skinfolds formula, that is based on Brazilian population data (15), as well as BMI according to international guidelines. BMI was calculated using the formula: BMI = (weight in kilograms)/(height in meters)^2^. Guedes 3-skinfolds formula is a body fat percentage estimation validated from a Brazilian population that uses the tricipital (TR), suprailiac (SI) and abdominal (AB) skinfolds in a body density formula: D = 1,17136 - 0,06706 log (TR + SI + AB), where “D” is the body density. With the “D” value calculated, we used the SIRI equation: BF% = [(4.95 / D) - 4.50] x 100, to obtain the BF% (15).

Cardiorespiratory fitness (CRF) was estimated by the 12 min-Cooper test, which is widely accepted as an indirect method for estimating the maximum oxygen consumption (VO_2_Max in mL·kg^−1^·min^−1^). The Cooper test is a running test in which the objective is to run as far as possible within 12 minutes. The distance reached is converted into oxygen consumption (VO_2_) using a validated formula (16). All tests were performed on the same athletic running track to improve the test’s precision and standardization among participants. For comparisons with other fire service studies, CRF was converted to metabolic equivalents (METs) by dividing VO_2_Max values by 3.5 (17).

Abdominal muscular endurance was assessed by the Sit-Up test that is a timed test in which the firefighter had to perform as many sit-up repetitions as possible in one minute, as suggested by the American College of Sports Medicine (ACSM) (18). Apart from CRF and body composition, the abdominal muscle evaluation (Sit-Up test) was included since muscular fitness is a health-related physical fitness component that should be trained to improve health (19) and because it was shown that core strength training could reduce injuries within firefighters (20).

### Obesity criteria and analysis

Obesity was defined using standard cut-off points for both indices: BF% > 25% and BMI ≥ 30 kg/m^2 ^(21). Comparisons between the two measures were determined using BF%-defined obesity as the reference measure. Agreement analysis was done by the following epidemiological indexes: 1) total agreement (TA), or accuracy, as the sum of the percentage of true positive (TP) and true negative (TN) values (TA = TP + TN); 2) BMI-sensitivity (sensitivity = [TP / (TP + FN)] x 100%), where FN is false negative; 3) BMI-specificity (specificity = [TN / (TN + FP)] x 100), where FP is false positive; 4); 4) Positive predictive value (PPV = [TP / (TP + FP) X 100]); 5) Negative predictive value (NPV = [TN / (TN + FN) X 100]); 6) Positive likelihood ratio (LR+ = [TP / (TP + FN)] / [FP / (FP + TN)]); Negative likelihood ratio (LR- = [FN / (TP + FN)] / [TN / (FP + TN)]) (22). All epidemiological indexes were calculated as their point value and 95% interval of confidence (95%IC).

In order to evaluate if there was any better BMI cut-off point different from the standardized one, we also employed the receiver operating characteristic (ROC) curve approach to analyze agreement and to explore different BMI cut-off points for this specific population.

Considering the possible influences of age and physical fitness on BMI, all agreement analyses were also performed stratifying participants by age, by CRF, using the CRF categories proposed by Baur and cols. (23), and by sit-up performance.

Because many variables were found to be non-normally distributed by skewness and kurtosis test for normality, we used the Cuzick nonparametric test for trend (24) and the area under the ROC curves were compared using the method proposed by Hanley and McNeil (25), with a Bonferoni *post-hoc* test. Pearson correlation coefficient between BMI and BF% was also calculated. Statistical significance was set as a two-tailed p value < 0.05. We used the IBM SPSS Statistics^®^ v17 (IBM Corporation, USA) and Stata 12.1 SE (Stata Corporation, College Station, TX) software packages for processing, analysis, and graphic design of the data.

## RESULTS

Mean age ± standard deviation (sd) of the participants was 37.4 ± 4.8 years old, varying from 24 to 49 years. The mean ± sd BMI was 26.5 ± 3.2 kg/m^2^, ranging from 17.6 to 41.4 kg/m^2^.

The overall characteristics of the study population in terms of age, CRF and sit-ups distribution, categorized by BMI subgroups, are shown on Table 1. There was a clear trend for a reduced physical fitness and an increase in age while BMI increases from its normal values (< 25.0 kg/m^2^) to the overweight and obese categories.

**Table 1:** Characteristics of the study population. Brazil, 3,822 male firefighters, 2009

	Body mass index			*P* trend^a^

	≤ 25 kg/m^2^	25–29 kg/m^2^	≥ 30 kg/m^2^	
N (row %)	1,274 (33.3)	2,038 (53.3)	510 (13.3)	
Age (yrs), mean (SD)	36.8 (5.0)	37.5 (4.7)	38.6 (4.6)	< 0.001
CRF (MET), mean (SD)	12.7 (1.7)	12.0 (1.7)	10.4 (1.5)	< 0.001
Sit-ups (rep), mean (SD)	27.8 (4.7)	27.4 (5.3)	25.3 (5.7)	< 0.001

CRF: cardiorespiratory fitness; BMI: body mass index; rep: repetitions; ^a ^nonparametric test for trend (Cuzick).

The overall prevalence of obesity estimated by BMI (13.3%) were similar to that obtained by BF% (15.9%), although BMI underestimated the prevalence of obesity in all analyzed categories. The overall difference between these estimates was -2.6%, varying across the CRF, sit-ups and age subgroups, but always smaller than -5.6%. However, the average relative differences were higher (-22.5%) and showed wide variation among subgroups (-2.9% // -65.9%) (Table 2).

**Table 2:** Relative prevalence of obesity by BF% and BMI in Brazil, 3,822 male firefighters, 2009

	Overall	CRF (MET)				Sit-ups (n)				Age (years)		
n	3,822	546	1,259	1,508	509	315	891	2,019	597	329	2,521	972
			
n %	100	14.3	32.9	39.5	13.3	8.2	23.3	52.8	15.6	8.6	66.0	25.4
			
		**< 10**	**10 - 12**	**> 12 - 14**	**> 14**	**≤ 30**	**30 - 40**	**41 - 50**	**> 50**	**20 - 30**	**31 - 40**	**41 - 50**

BF% (%)	15.9	41.6	19.9	7.2	4.1	36.8	21.5	12.9	6.5	9.1	16.1	17.5
BMI (%)	13.3	38.8	17.2	4.9	1.4	31.4	18.1	10.9	4.9	5.8	12.9	17.0
Δ Absolute	-2.6	-2.8	-2.7	-2.3	-2.7	-5.4	-3.4	-2.0	-1.6	-3.3	-3.2	-0.5
Δ Relative	-16.4	-6.7	-13.6	-31.9	-65.9	-14.7	-15.8	-15.5	-24.6	-36.3	-19.9	-2.9

CRF: cardiorespiratory fitness; BF%: body fat percentage; BMI: body mass index; Δ: variation.

**Table 3:** Agreement between BMI and body fat percentage for defining obesity in Brazil, 3,822 male firefighters, 2009

			Body fat % > 25%				Agreement: 85.8% (95%CI 84.7–86.9) Sensitivity: 47.4% (95%CI 43.4–51.5) Specificity: 93.1% (95%CI 92.2–93.9) Positive likelihood ratio: 6.87 (95%CI 5.90–8.00) Negative likelihood ratio: 0.56 (95%CI 0.52–0.61) Positive predictive value: 56.5% (95%CI 52.0–60.8) Negative predictive value: 90.4% (95%CI 89.3–91.4)		
			Yes	No		Total			
			N (%)	N (%)		N			
BMI ≥ 30 kg/m^2^	Yes	N (%)	288 (7.5%)	222 (5.8%)		510			
	No	N (%)	319 (8.3%)	2,993 (78.3%)		3,312			
	Total	*N*	607	3,215		3,822			
Alternative cut-offs									
Cut-off					27 kg/m^2^			28 kg/m^2^	29 kg/m^2^
Agreement, % (95%CI)					69.9 (68.5–71.4)			77.7 (76.4–79.0)	82.9 (81.7–84.1)
Sensitivity, % (95%CI)					82.4 (79.1–85.3)			72.2 (68.4–75.7)	60.5 (56.4–64.4)
Specificity, % (95%CI)					67.6 (65.9–69.2)			78.8 (77.3–80.2)	87.2 (86.0–88.3)
Positive likelihood ratio, (95%CI)					2.54 (2.39–2.70)			3.40 (3.13–3.70)	4.72 (4.22–5.27)
Negative likelihood ratio, (95%CI)					0.26 (0.22–0.31)			0.35 (0.31–0.40)	0.45 (0.41–0.50)
Positive predictive value, % (95%CI)					32.4 (30.1–34.8)			39.1 (36.2–42.0)	47.1 (43.6–50.7)
Negative predictive value, % (95%CI)					95.3 (94.4–96.1)			93.7 (92.8–94.6)	92.1 (91.1–93.0)

We observed a high total agreement: 85.8% (95%CI: 84.7 – 86.9), with good specificity and poor sensitivity: 93.1% (95%CI: 92.2 – 93.9) and 47.4% (95%CI: 43.4 – 51.5), respectively. Furthermore, the agreement varied significantly with the variance in obesity prevalence in different age and physical fitness subgroups (Tables 3-6).

**Table 4:** Diagnosis of obesity (body fat percentage ≥ 25) based on body mass index. Analysis stratified by cardiorespiratory fitness (n = 3,822 male firefighters)

	Cardiorespiratory fitness (METs)			

	< 10 *N* = 546	10-12 *N* = 1,259	> 12–14 *N* = 1,508	> 14 *N* = 509
**BMI ≥ 27 kg/m^**2**^**				

Agreement, % (95%CI)	61.9 (57.7–66.0)	63.8 (61.1–66.4)	73.2 (70.9–75.4)	84.1 (80.6–87.2)
Sensitivity, % (95%CI)	89.9 (85.2–93.5)	85.3 (80.3–89.4)	70.4 (60.8–78.8)	28.6 (11.3–52.2)
Specificity, % (95%CI)	42.0 (36.5–47.6)	58.4 (55.3–61.5)	73.4 (71.0–75.7)	86.5 (83.1–89.4)
Positive likelihood ratio, (95%CI)	1.55 (1.40–1.72)	2.05 (1.88–2.24)	2.65 (2.28–3.08)	2.11 (1.04–4.31)
Negative likelihood ratio, (95%CI)	0.24 (0.16–0.36)	0.25 (0.19–0.34)	0.40 (0.30–0.54)	0.83 (0.63–1.09)
Positive predictive value, % (95%CI)	52.4 (47.3–57.5)	33.8 (30.1–37.6)	17.0 (13.6–20.8)	8.3 (3.1–17.3)
Negative predictive value, % (95%CI)	85.4 (78.8–90.5)	94.1 (91.9–95.8)	97.0 (95.8–97.9)	96.6 (94.4–98.1)

**BMI ≥ 28 kg/m^**2**^**				

Agreement, % (95%CI)	69.0 (65.0–72.9)	70.9 (68.3–73.4)	82.5 (80.5–84.4)	89.8 (86.8–92.3)
Sensitivity, % (95%CI)	85.5 (80.2–89.8)	72.5 (66.5–77.9)	54.6 (44.8–64.2)	14.3 (3.0–36.3)
Specificity, % (95%CI)	57.4 (51.7–62.9)	70.5 (67.6–73.3)	84.6 (82.6–86.5)	93.0 (90.4–95.1)
Positive likelihood ratio, (95%CI)	2.00 (1.75–2.30)	2.46 (2.18–2.78)	3.56 (2.88–4.39)	2.05 (0.68–6.14)
Negative likelihood ratio, (95%CI)	0.25 (0.18–0.35)	0.39 (0.32–0.48)	0.54 (0.43–0.66)	0.92 (0.77–1.10)
Positive predictive value, % (95%CI)	58.8 (53.3–64.2)	38.0 (33.6–42.5)	21.5 (16.8–26.9)	8.1 (1.7–21.9)
Negative predictive value, % (95%CI)	84.7 (79.2–89.2)	91.2 (88.9–93.1)	96.0 (94.8–97.0)	96.2 (94.0–97.7)

**BMI ≥ 29 kg/m^**2**^**				

Agreement, % (95%CI)	74.2 (70.3–77.8)	76.5 (74.0–78.8)	87.9 (86.1–89.5)	93.7 (91.2–95.7)
Sensitivity, % (95%CI)	77.5 (71.5–82.8)	58.2 (51.8–64.3)	39.8 (30.5–49.7)	9.5 (1.2–30.4)
Specificity, % (95%CI)	71.8 (66.5–76.7)	81.1 (78.5–83.4)	91.6 (90.0–93.0)	97.3 (95.5–98.6)
Positive likelihood ratio, (95%CI)	2.75 (2.28–3.32)	3.07 (2.60–3.62)	4.72 (3.54–6.31)	3.58 (0.86–14.8)
Negative likelihood ratio, (95%CI)	0.31 (0.24–0.40)	0.52 (0.44–0.60)	0.66 (0.56–0.77)	0.93 (0.81–1.07)
Positive predictive value, % (95%CI)	66.2 (60.1–71.8)	43.3 (38.0–48.8)	26.7 (20.1–34.2)	13.3 (1.7–40.5)
Negative predictive value, % (95%CI)	81.8 (76.8–86.1)	88.6 (86.4–90.6)	95.2 (93.9–96.3)	96.2 (94.1–97.7)

**BMI ≥ 30 kg/m^**2**^**				

Agreement, % (95%CI)	75.3 (71.4–78.8)	80.6 (78.3–82.8)	91.1 (89.6–92.5)	94.5 (92.1–96.3)
Sensitivity, % (95%CI)	67.0 (60.4–73.0)	44.6 (38.4–51.0)	22.2 (14.8–31.2)	0.0 (0.0–16.1)
Specificity, % (95%CI)	81.2 (76.5–85.3)	89.6 (87.5–91.4)	96.4 (95.3–97.3)	98.6 (97.1–99.4)
Positive likelihood ratio, (95%CI)	3.56 (2.78–4.55)	4.28 (3.41–5.38)	6.22 (3.98–9.72)	1.48^a^ (0.09–25.1)
Negative likelihood ratio, (95%CI)	0.41 (0.34–0.49)	0.62 (0.55–0.69)	0.81 (0.73–0.89)	0.99^a^ (0.93–1.06)
Positive predictive value, % (95%CI)	71.7 (65.1–77.7)	51.6 (44.8–58.4)	32.4 (22.0–44.3)	0.0 (0.0–41.0)
Negative predictive value, % (95%CI)	77.5 (72.7–81.9)	86.7 (84.4–88.7)	94.1 (92.8–95.3)	95.8 (93.7–97.4)

^a ^Values estimated using the substitution formula (0.5 added to all cell frequencies).

**Table 5:** Diagnosis of obesity (body fat percentage ≥ 25) based on body mass index. Analysis stratified by number of sit-ups (n = 3,822 male firefighters)

	Completed sit-ups (n)			

	≤ 30 *N* = 315	31–40 *N* = 891	41–50 *N* = 2,019	> 50 *N* = 597
**BMI ≥ 27 kg/m^**2**^**				

Agreement, % (95%CI)	68.3 (62.8–73.4)	69.0 (65.9–72.0)	68.5 (66.4–70.5)	77.1 (73.5–80.4)
Sensitivity, % (95%CI)	87.9 (80.6–93.2)	87.0 (81.4–91.4)	78.5 (73.0–83.3)	69.2 (52.4–83.0)
Specificity, % (95%CI)	56.8 (49.6–63.8)	64.1 (60.4–67.7)	67.0 (64.8–69.2)	77.6 (73.9–81.0)
Positive likelihood ratio, (95%CI)	2.03 (1.71–2.42)	2.42 (2.16–2.71)	2.38 (2.17–2.61)	3.09 (2.38–4.01)
Negative likelihood ratio, (95%CI)	0.21 (0.13–0.35)	0.20 (0.14–0.29)	0.32 (0.25–0.41)	0.40 (0.25–0.64)
Positive predictive value, % (95%CI)	54.3 (46.8–61.5)	40.0 (35.2–44.8)	26.0 (23.0–29.2)	17.8 (12.0–24.8)
Negative predictive value, % (95%CI)	89.0 (82.2–93.8)	94.7 (92.3–96.6)	95.5 (94.2–96.6)	97.3 (95.3–98.6)

**BMI ≥ 28 kg/m^**2**^**				

Agreement, % (95%CI)	72.7 (67.4–77.5)	75.3 (72.3–78.1)	77.5 (75.6–79.3)	84.8 (81.6–87.5)
Sensitivity, % (95%CI)	80.2 (71.7–87.0)	76.0 (69.4–81.9)	68.8 (62.8–74.4)	51.3 (34.8–67.6)
Specificity, % (95%CI)	68.3 (61.4–74.7)	75.1 (71.7–78.3)	78.8 (76.8–80.7)	87.1 (84.0–89.8)
Positive likelihood ratio, (95%CI)	2.53 (2.03–3.17)	3.05 (2.63–3.55)	3.25 (2.87–3.67)	3.97 (2.73–5.78)
Negative likelihood ratio, (95%CI)	0.29 (0.20–0.42)	0.32 (0.25–0.41)	0.39 (0.33–0.47)	0.56 (0.40–0.77)
Positive predictive value, % (95%CI)	59.6 (51.5–67.4)	45.6 (40.1–51.3)	32.4 (28.5–36.5)	21.7 (13.8–31.6)
Negative predictive value, % (95%CI)	85.5 (79.1–90.6)	91.9 (89.4–94.0)	94.5 (93.2–95.6)	96.2 (94.2–97.7)

**BMI ≥ 29 kg/m^**2**^**				

Agreement, % (95%CI)	77.1 (72.1–81.7)	79.9 (77.1–82.5)	83.1 (81.4–84.7)	89.9 (87.3–92.2)
Sensitivity, % (95%CI)	74.1 (65.2–81.8)	64.1 (56.8–70.8)	54.6 (48.3–60.8)	41.0 (25.6–57.9)
Specificity, % (95%CI)	78.9 (72.6–84.3)	84.3 (81.3–86.9)	87.3 (85.7–88.8)	93.4 (91.0–95.3)
Positive likelihood ratio, (95%CI)	3.51 (2.63–4.69)	4.07 (3.33–4.98)	4.31 (3.65–5.08)	6.19 (3.80–10.1)
Negative likelihood ratio, (95%CI)	0.33 (0.24–0.45)	0.43 (0.35–0.52)	0.52 (0.45–0.60)	0.63 (0.49–0.82)
Positive predictive value, % (95%CI)	67.2 (58.3–75.2)	52.8 (46.2–59.3)	38.9 (33.9–44.1)	30.2 (18.3–44.3)
Negative predictive value, % (95%CI)	84.0 (77.9–88.9)	89.5 (86.9–91.7)	92.9 (91.5–94.1)	95.8 (93.7–97.3)

**BMI ≥ 30 kg/m^**2**^**				

Agreement, % (95%CI)	78.1 (73.1–82.5)	82.2 (79.5–84.6)	86.6 (85.0–88.0)	93.0 (90.6–94.9)
Sensitivity, % (95%CI)	62.9 (53.5–71.7)	50.5 (43.2–57.8)	40.4 (34.4–46.6)	33.3 (19.1–50.2)
Specificity, % (95%CI)	86.9 (81.4–91.3)	90.8 (88.5–92.9)	93.4 (92.1–94.5)	97.1 (95.4–98.4)
Positive likelihood ratio, (95%CI)	4.82 (3.28–7.08)	5.52 (4.20–7.24)	6.12 (4.87–7.70)	11.6 (6.03–22.4)
Negative likelihood ratio, (95%CI)	0.43 (0.33–0.54)	0.71 (0.67–0.74)	0.64 (0.58–0.71)	0.69 (0.55–0.86)
Positive predictive value, % (95%CI)	73.7 (63.9–82.1)	60.2 (52.2–67.9)	47.5 (40.8–54.3)	44.8 (26.4–64.3)
Negative predictive value, % (95%CI)	80.1 (74.1–85.2)	87.0 (84.3–89.3)	91.4 (90.0–92.6)	95.4 (93.4–97.0)

**Figure 1:**
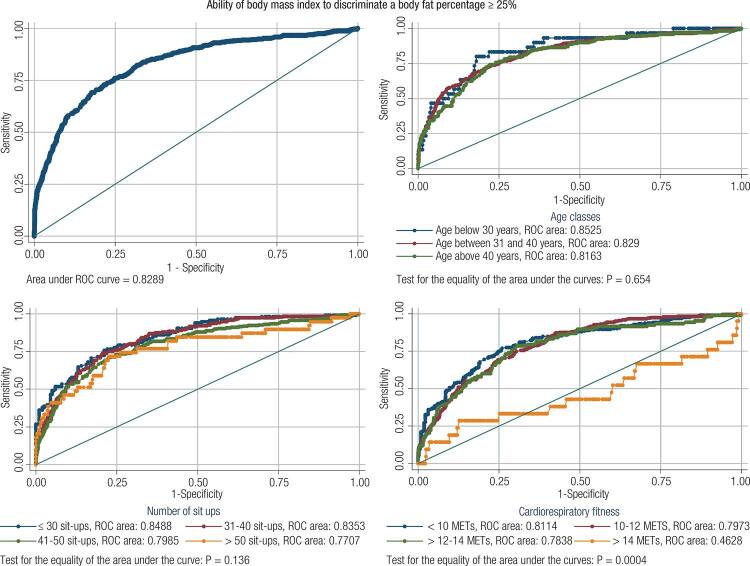
Non-parametric ROC curves showing the ability of BMI to identity obesity (≥ 30 kg/m2) as compared to BF% (> 25%) within 3,822 male military firefighters (upper left panel) and stratified by age (upper right), by the performance on sit up test (bottom left) and by CRF.

The ROC curves for BMI to detect BF%-defined obesity showed an overall area under the curve (AUC) equal to 0.83. When stratified by age, all age-category AUC were similar (p = 0.65) and above 0.82. The same profile was observed for the sit-up stratification, in which the sit-up-categories AUC ranged from 0.77 to 0.85, with no differences among categories (p = 0.14). After stratifying by CRF, the ROC curves analysis showed a low AUC (0.46) for those with the highest CRF (> 14 MET) that was statistically different from all others CRF categories (AUC ≥ 0.78; p < 0.05) but similar within them (p > 0.05) (Figure 1). Further information of the BMI diagnostic performance, using the standardized and alternative cut-off points are shown on Tables 2-6.

**Table 6:** Diagnosis of obesity (body fat percentage ≥ 25) based on body mass index. Analysis stratified by age class (n = 3,822 male firefighters)

	Age (completed years)		

	≤ 30 years *N* = 329	31–40 years *N* = 2,521	> 40 years *N* = 972
**BMI ≥ 27 kg/m^**2**^**			

Agreement, % (95%CI)	81.2 (76.5–85.2)	69.6 (67.8–71.4)	67.0 (63.9–69.9)
Sensitivity, % (95%CI)	80.0 (61.4–92.3)	81.6 (77.5–85.2)	84.7 (78.4–89.8)
Specificity, % (95%CI)	81.3 (76.4–85.5)	67.3 (65.3–69.3)	63.2 (59.8–66.6)
Positive likelihood ratio, (95%CI)	4.27 (3.18–5.74)	2.50 (2.31–2.69)	2.30 (2.06–2.57)
Negative likelihood ratio, (95%CI)	0.25 (0.12–0.50)	0.27 (0.22–0.34)	0.24 (0.17–0.35)
Positive predictive value, % (95%CI)	30.0 (20.3–41.3)	32.5 (29.6–35.4)	32.8 (28.4–37.4)
Negative predictive value, % (95%CI)	97.6 (94.8–99.1)	95.0 (93.8–96.0)	95.1 (92.9–96.8)

**BMI ≥ 28 kg/m^**2**^**			

Agreement, % (95%CI)	85.7 (81.5–89.3)	77.9 (76.2–79.5)	74.6 (71.7–77.3)
Sensitivity, % (95%CI)	53.3 (34.3–71.7)	71.7 (67.1–76.1)	76.5 (69.4–82.6)
Specificity, % (95%CI)	89.0 (84.9–92.3)	79.1 (77.3–80.8)	74.2 (71.0–77.2)
Positive likelihood ratio, (95%CI)	4.83 (3.04–7.69)	3.43 (3.10–3.80)	2.96 (2.57–3.42)
Negative likelihood ratio, (95%CI)	0.52 (0.36–0.77)	0.36 (0.31–0.42)	0.32 (0.24–0.42)
Positive predictive value, % (95%CI)	32.7 (19.9–47.5)	39.8 (36.2–43.4)	38.6 (33.4–44.0)
Negative predictive value, % (95%CI)	95.0 (91.8–97.2)	93.6 (92.3–94.7)	93.7 (91.5–95.5)

**BMI ≥ 29 kg/m^**2**^**			

Agreement, % (95%CI)	89.4 (85.5–92.5)	83.5 (82.0–84.9)	79.4 (76.7–81.9)
Sensitivity, % (95%CI)	46.7 (28.3–65.7)	59.5 (54.5–64.3)	65.3 (57.6–72.4)
Specificity, % (95%CI)	93.6 (90.3–96.1)	88.1 (86.6–89.4)	82.4 (79.6–85.0)
Positive likelihood ratio, (95%CI)	7.34 (4.11–13.1)	4.99 (4.33–5.74)	3.71 (3.08–4.47)
Negative likelihood ratio, (95%CI)	0.57 (0.41–0.80)	0.46 (0.41–0.52)	0.42 (0.34–0.52)
Positive predictive value, % (95%CI)	42.4 (25.5–60.8)	49.0 (44.5–53.5)	44.0 (37.8–50.4)
Negative predictive value, % (95%CI)	94.6 (91.4–96.9)	91.9 (90.6–93.0)	91.8 (89.6–93.7)

**BMI ≥ 30 kg/m^**2**^**			

Agreement, % (95%CI)	91.2 (87.6–94.0)	86.6 (85.2–87.9)	82.2 (79.6–84.6)
Sensitivity, % (95%CI)	33.3 (17.3–52.8)	48.4 (43.5–53.4)	47.6 (39.9–55.4)
Specificity, % (95%CI)	97.0 (94.4–98.6)	93.9 (92.8–94.9)	89.5 (87.2–91.6)
Positive likelihood ratio, (95%CI)	11.1 (4.88–25.1)	7.93 (6.53–9.64)	4.55 (3.52–5.88)
Negative likelihood ratio, (95%CI)	0.69 (0.56–0.74)	0.55 (0.50–0.60)	0.58 (0.51–0.68)
Positive predictive value, % (95%CI)	52.6 (28.9–75.6)	60.4 (54.9–65.8)	49.1 (41.2–57.0)
Negative predictive value, % (95%CI)	93.5 (90.2–96.0)	90.4 (89.1–91.6)	89.0 (86.6–91.0)

**Figure 2:**
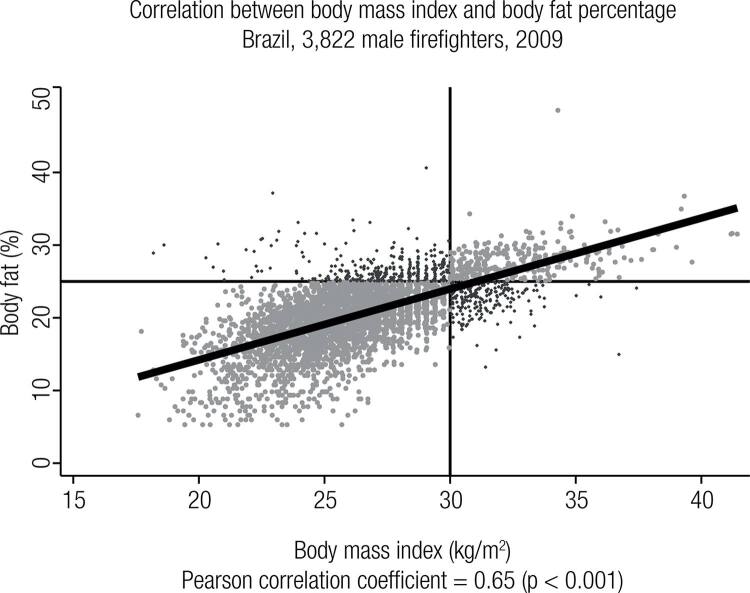
Correlation between BMI and BF% values in 3,822 military male firefighters. The chart shows quadrants defined by the standard cut-off points (BMI ≥ 30 kg/m2 and BF% > 25%).

A significant correlation between BMI and BF% values was found (r = 0.65; p < 0.001), as shown on Figure 2 in which BMI misclassification is represented by the darker dots.

## DISCUSSION

In this large military firefighter cohort we showed that both BMI and BF% measures yielded similar overall obesity prevalences. Similar results were observed in each age and physical fitness subgroup as well. Also important is the high total agreement between both measures in all groups, ranging from 75.2% to 94.8%.

Contrary to common concerns, BMI did not overestimate obesity prevalence. In fact, BMI actually underestimated obesity in all groups. This BMI-defined obesity underestimation trend has been also reported on several studies with different populations pooled on a meta-analysis published in 2010 (26). This tendency has been an interesting finding among firefighters, since they are supposedly more fit than the general population (27,28).

Poston and cols. also observed a BMI-defined obesity underestimation trend among US firefighters (29). It should be noted that even with the same underestimation tendency, the obesity prevalence reported by Poston and cols. in US firefighters was very high (25.3% to 35.6%) (29,30), as compared to the one observed in this Brazilian cohort. A great distinction between the Brazilian and the US Fire Departments is that the former one is a military institution, with specific annual physical training demands and requirements (31) that may explain part of these differences, whereas the latter is a civil organization with variable training regimens, if any, and rarely applying strict physical fitness requirements.

As regard to the possible BMI-misclassification, we should consider that the BMI inherent incapacity to distinguish lean mass from body fat composition is probably higher on intermediate BMI values (from 25.0 to 30.0 kg/m^2^), as pointed out elsewhere either for the general population (32) and for specific groups as coronary heart disease patients (33). It should also be considered that the BMI-misclassification occurred not only on the high values of BMI, as usually supposed. Figure 2 shows that there were some volunteers with low BMI values (< 30 kg/m^2^) but with a high (> 25%) body fat. Again, most of these cases occurred on intermediate BMI values, specifically between 27.0 to 30.0 kg/m^2^. Our data reinforce this hypothesis once the BMI-sensitivity increases while its specificity decreases when the cut-off point is changed from 30.0 to 27.0 kg/m^2^, which means that with lower cut-off points some subject with BF% < 25% would be considered to be obese.

Furthermore, almost all indices were affected by CRF, sit-ups and age, with better sensitivity in the less fit (CRF and sit-ups) and in the older groups. On the other hand, specificity was highest among the fittest and youngest groups (Table 2). This trend was observed in all analyzed cut-off points (Tables 3-5). The same trend was also observed for the likelihood ratios, where LR+ tends to increase while cut-off point increases from 27.0 kg/m^2^ to the standardized 30.0 kg/m^2 ^and among better physical fitness and younger groups as compared to those with lower physical fitness and higher age. As regard to the LR- we observed exactly the opposite. LR- values were worse (closer to one) on lower cut-off points and among younger and less fit firefighters while it tended to zero (better values) when the sensitivity tended to be higher, i.e., in lower cut-off points and among the older and less fit groups.

Besides, we observed very low true positive percentages and very high true negative percentages. Despite a very good total agreement, BMI capacity to correctly identify obesity as compared to BF% was very low. On the other hand, BMI was a very good tool to exclude BF%-defined obesity.

Another important finding is that even with the good accuracy observed in all analyzed subgroups, ROC curve stratified by CRF (Figure 1) showed that BMI was not good enough to identify firefighters with BF% > 25% within those with CRF > 14 MET. The reduced AUC (0.46) observed among firefighters with CRF > 14 METs means that BMI was not a helpful tool to identify obesity in this specific subgroup (22). These results reinforce the idea that BMI cut-off points should consider some subgroup specificities, as the CRF itself or the ethnicity, as already proposed by the World Health Organization for the Asian populations (34).

In our analysis, a better agreement was seen when the standardized cut-off point was used. However, it should be highlighted that the high agreement was almost dependent on the high specificity, once the percentage of true positive was very low. When a good sensitivity was obtained (cut-off = 27.0 kg/m^2^), the total agreement drops 10% or more, either for the whole population or for any subgroup (Tables 2-5).

While this study has been done with a large firefighter cohort, there are some limitations that must be considered. First, we compared BMI against a reference method (BF% by skinfold thickness) that is not considered the gold-standard one for body composition assessment. However, we aimed to compare BMI performance against a method that is largely employed worldwide and more suitable for large sample studies than the DEXA, apart from the fact that the estimation of BF% by skinfold thickness has long been recognized as a feasible, valid and low cost method (18,35). Besides the limitations of considering BF% as a reference method, it should be taken into account that BF% estimated by skinfold thickness overcomes the inability of BMI to distinguish lean mass from body fat, which is the BMI most important limitation. Furthermore, Okorodudu and cols. study shows that when BMI obesity diagnostic performance was analyzed considering only the most precise techniques to evaluate body fat, their agreement analysis didn’t change significantly (26).

Finally, it is also important to consider that some of the volunteers could have performed bellow their maximum capacity since they knew in advance the minimum threshold that they would need to reach in order to achieve success on the annual physical evaluation. The very high consistency of our data shows that this possibility has probably not affected the results.

In conclusion, this cross-sectional study conducted among a large physically active population showed that BMI and body fat percentage yielded similar obesity prevalence in the whole sample and in each subgroup stratified per age, sit-up and CRF performance. We also observed a very high index of total agreement. Contrary to common concerns, BMI did not overestimate obesity prevalence, even among the fittest subgroup. However, ROC curve analysis demonstrated that BMI standardized cut-off point was not useful to identify obesity in the group with CRF > 14 METs. BMI ≥ 30 km/m^2^ showed to be highly specific as a screen to exclude obesity in this large firefighter sample, but it resulted on low sensitivity. Because of systematic underestimation, a lower BMI cut point might be considered in this and other physically active populations

### Practical applications

Considering its ease of measurement, low cost and the high total agreement of standardized BMI cut-off point as compared to BF%-obesity definitions, BMI is an excellent screening tool to estimate obesity prevalence in this physically active population. Standardized BMI cut-off point was less useful to identify obesity in the fittest group (CRF >14 METs). Because of systematic obesity underestimation, a lower BMI cut-off point (27.0 kg/m^2^) might be considered in this and other physically active populations, especially for obesity prevention programs, when BMI-sensitivity must be emphasized. Our results reinforce the idea that BMI cut-off points should consider some specific characteristics, as age and physical fitness. Our findings are likely generalizable to other similar active populations as such, law enforcement and armed forces professionals.

## References

[1] .von Ruesten A, Steffen A, Floegel A, van der A DL, Masala G, Tjønneland A, et al.Trend in obesity prevalence in European adult cohort populations during follow-up since 1996 and their predictions to 2015. PLoS One. 2011;6(11):e27455. 10.1371/journal.pone.0027455 PMC3213129 22102897

[2] .Flegal KM, Carroll MD, Kit BK, Ogden CL. Prevalence of obesity and trends in the distribution of body mass index among US adults, 1999-2010. JAMA. 2012;307(5):491-7. 10.1001/jama.2012.39 22253363

[3] .Flegal KM, Carroll MD, Kuczmarski RJ, Johnson CL. Overweight and obesity in the United States: prevalence and trends, 1960-1994. Int J Obes Relat Metab Disord. 1998;22(1):39-47. 10.1038/sj.ijo.0800541 9481598

[4] .Misra A, Khurana L. Obesity and the metabolic syndrome in developing countries. J Clin Endocrinol Metab. 2008;93(11 Suppl 1):S9-30. 10.1210/jc.2008-1595 18987276

[5] .World Health Organization – WHO. World Health Organization Health Statistics 2012. Available at: http://apps.who.int/iris/bitstream/10665/44844/1/9789241564441_eng.pdf?ua=1. Accessed on: Jan 6, 2016.

[6] .Sakurai Y, Teruya K, Shimada N, Umeda T, Tanaka H, Muto T, et al. Association between duration of obesity and risk of non-insulin-dependent diabetes mellitus. The Sotetsu Study. Am J Epidemiol. 1999;149(3):256-60. 10.1093/oxfordjournals.aje.a009800 9927221

[7] .Landsberg L, Aronne LJ, Beilin LJ, Burke V, Igel LI, Lloyd-Jones D, et al. Obesity-related hypertension: pathogenesis, cardiovascular risk, and treatment: a position paper of The Obesity Society and the American Society of Hypertension. J Clin Hypertens (Greenwich). 2013;15(1):14-33. 10.1111/jch.12049 PMC8108268 23282121

[8] .Australian Government – National Health and Medical Research Council – ANHMR. Clinical Practice Guidelines for the Management of Overweight and Obesity in Adults. 2013. Available at: https://www.nhmrc.gov.au/_files_nhmrc/publications/attachments/n57_obesity_guidelines_140630.pdf. Accessed on: Jan 6, 2016.

[9] .Erselcan T, Candan F, Saruhan S, Ayca T. Comparison of body composition analysis methods in clinical routine. Ann Nutr Metab. 2000;44(5-6):243-8. 10.1159/000046691 11146331

[10] .Associação Brasileira para o Estudo da Obesidade e da Síndrome Metabólica – Diretrizes Brasileiras de Obesidade 2009/2010 – ABESO, 2009. (Portuguese: Brazilian Association for the Study of Obesity and Metabolic Syndrome – Brazilian guidelines for obesity 2009/2010). Available at: http://www.abeso.org.br/pdf/diretrizes_brasileiras_obesidade_2009_2010_1.pdf. Accessed on: Jan 6, 2016.

[11] .National Institutes of Healh – National Heart, Lung, and Blood Institute and North American Association for the Study of Obesity. The Practical Guide Identification, Evaluation, and Treatment of Overweight and Obesity in Adults. 2000. Available at: http://www.nhlbi.nih.gov/files/docs/guidelines/prctgd_c.pdf. Accessed on: Jan 6, 2016.

[12] .Centers for Disease Control and Prevention – CDC. National Health and Nutrition Examination Survey – NHANES 2013/14. Available at: http://www.cdc.gov/nchs/nhanes.htm. Accessed on: Jan 6, 2016.

[13] .Iser BP, Yokota RT, de Sá NN, de Moura L, Malta DC. [Protection from chronic diseases and the prevalence of risk factors in Brazilian state capitals--main results from Vigitel 2010]. Cien Saude Colet. 2012;17(9):2343-56. 10.1590/s1413-81232012000900015 22996885

[14] .Fact sheets from the Surgeon General’s Call to Action to Prevent and Decrease Overweight and Obesity. W V Med J. 2002;98(6):234-43. 12645272

[15] .Guedes DP. Composição corporal: princípios, técnicas e aplicações. Londrina: APEF; 1994.

[16] .Cooper KH. A means of assessing maximal oxygen intake. Correlation between field and treadmill testing. JAMA. 1968;203(3):201-4. 5694044

[17] .Garber CE, Blissmer B, Deschenes MR, Franklin BA, Lamonte MJ, Lee IM, et al. American College of Sports Medicine position stand. Quantity and quality of exercise for developing and maintaining cardiorespiratory, musculoskeletal, and neuromotor fitness in apparently healthy adults: guidance for prescribing exercise. Med Sci Sports Exerc. 2011;43(7):1334-59. 10.1249/MSS.0b013e318213fefb 21694556

[18] .American College of Sports Medicine – ACSM. ACSM’s guidelines for exercise testing and prescription. Baltimore: Lippincott Williams & Wilkins; 2006.

[19] .Haskell WL, Lee IM, Pate RR, Powell KE, Blair SN, Franklin BA, et al. Physical activity and public health: updated recommendation for adults from the American College of Sports Medicine and the American Heart Association. Med Sci Sports Exerc. 2007;39(8):1423-34. 10.1249/mss.0b013e3180616b27 17762377

[20] .Peate WF, Bates G, Lunda K, Francis S, Bellamy K. Core strength: a new model for injury prediction and prevention. J Occup Med Toxicol. 2007;2:3. 10.1186/1745-6673-2-3 PMC1865378 17428333

[21] .World Health Organization – WHO. Physical status: the use and interpretation of anthropometry. Report of a WHO Expert Committee. World Health Organ Tech Rep Ser. 1995;854:1-452. Available at: http://www.who.int/childgrowth/publications/physical_status/en/. Accessed on: Jan 6, 2016. 8594834

[22] .Fletcher RH, Fletcher SW. Clinical epidemiology: the essentials. Philadelphia: Lippincott Williams & Wilkins, 2005.

[23] .Baur DM, Christophi CA, Kales SN. Metabolic syndrome is inversely related to cardiorespiratory fitness in male career firefighters. J Strength Con Res. 2012;26(9):2331-7. 10.1519/JSC.0b013e31823e9b19 22067249

[24] .D’Agostino RB, Belanger AJ, D’Agostino Jr RB. A suggestion for using powerful and informative tests of normality. American Statistician. 1990;44:316-21.

[25] .Hanley JA, McNeil BJ. A method of comparing the areas under receiver operating characteristic curves derived from the same cases. Radiology. 1983;148(3):839-43. 10.1148/radiology.148.3.6878708 6878708

[26] .Okorodudu DO, Jumean MF, Montori VM, Romero-Corral A, Somers VK, Erwin PJ, et al. Diagnostic performance of body mass index to identify obesity as defined by body adiposity: a systematic review and meta-analysis. Int J Obes (Lond). 2010;34(5):791-9. 10.1038/ijo.2010.5 20125098

[27] .International Association of Fire Fighters – IAFF. The Fire Service Joint Labor Management Wellness-Fitness Initiative (2008). Available at: http://www.iaff.org/hs/lodd_manual/resources/iaff-iafcwfi3rdedition.pdf. Accessed on: July 6, 2015.

[28] .Choi B, Schnall P, Dobson M, Israel L, Landsbergis P, Galassetti P, et al. Exploring occupational and behavioral risk factors for obesity in firefighters: a theoretical framework and study design. Saf Health Work. 2011;2(4):301-12. 10.5491/SHAW.2011.2.4.301 PMC3430916 22953214

[29] .Poston WS, Haddock CK, Jahnke SA, Jitnarin N, Tuley BC, Kales SN. The prevalence of overweight, obesity, and substandard fitness in a population-based firefighter cohort. J Occup Environ Med. 2011;53(3):266-73. 10.1097/JOM.0b013e31820af362 PMC5826653 21386691

[30] .Poston WS, Haddock CK, Jahnke SA, Jitnarin N, Day RS. An examination of the benefits of health promotion programs for the national fire service. BMC Public Health. 2013;13:805. 10.1186/1471-2458-13-805 PMC3846399 24007391

[31] .Nogueira EC, Porto LG, Nogueira RM, Martins WR, Fonseca RM, Lunardi CC, et al. Body Composition is Strongly Associated With Cardiorespiratory Fitness in a Large Brazilian Military Firefighter Cohort: The Brazilian Firefighters Study. J Strength Cond Res. 2016;30(1):33-8. 10.1519/JSC.0000000000001039 26691405

[32] .Romero-Corral A, Somers VK, Sierra-Johnson J, Thomas RJ, Collazo-Clavell ML, Korinek J, et al. Accuracy of body mass index in diagnosing obesity in the adult general population. Int J Obes (Lond). 2008;32(6):959-66. 10.1038/ijo.2008.11 PMC2877506 18283284

[33] .Piers LS, Soares MJ, Frandsen SL, O’Dea K. Indirect estimates of body composition are useful for groups but unreliable in individuals. Int J Obes Relat Metab Disord. 2000;24(9):1145-52. 10.1038/sj.ijo.0801387 11033983

[34] .World Health Organization – WHO. World Health Organization/International Association for the Study of Obesity/International Obesity Task Force. The Asia-Pacific perspective: redefining obesity and its treatment. 2000. Available at: http://www.wpro.who.int/nutrition/documents/Redefining_obesity/en/. Accessed on: Jan 6, 2016.

[35] .Himes JH, Roche AF, Siervogel RM. Compressibility of skinfolds and the measurement of subcutaneous fatness. Am J Clin Nutr. 1979;32(8):1734-40. 10.1093/ajcn/32.8.1734 463811

